# Trends in hepatitis B virus testing practices and management in HIV clinics across sub-Saharan Africa

**DOI:** 10.1186/s12879-017-2768-z

**Published:** 2017-11-01

**Authors:** Patrick A. Coffie, Matthias Egger, Michael J. Vinikoor, Marcel Zannou, Lameck Diero, Akouda Patassi, Mark H. Kuniholm, Moussa Seydi, Guillaume Bado, Ponsiano Ocama, Monique I. Andersson, Eugène Messou, Albert Minga, Philippa Easterbrook, Kathryn Anastos, François Dabis, Gilles Wandeler, for the IeDEA collaboration

**Affiliations:** 1 Programme PACCI, CHU Treichville, Site de Recherche ANRS, Abidjan, Côte d’Ivoire; 2 Département de Dermatologie et d’Infectiologie, UFR des Sciences Médicales, Université Félix Houphouët Boigny, Abidjan, Côte d’Ivoire; 3 Institute of Social and Preventive Medicine, University of Bern, Bern, Switzerland; 4 Centre for Infectious Disease Epidemiology and Research (CIDER), University of Cape Town, Cape Town, South Africa; 5 Centre for Infectious Disease Research in Zambia, Lusaka, Zambia; 6 Department of Medicine at University of Alabama, Birmingham, AL USA; 7 Service de Médecine Interne, CNHU Hubert Maga, Cotonou, Benin; 8 Department of Medicine, Moi University, College of Health Sciences, School of Medicine, Eldoret, Kenya; 9 Service des Maladies Infectieuses et de Pneumologie, CHU Sylvanus Olympio, Lomé, Togo; 10 Department of Epidemiology and Biostatistics, University at Albany, State University of New York, Rensselaer, NY USA; 11 Department of Infectious Diseases, Fann University Hospital, Dakar, Senegal; 12 Hôpital de Jour, Service des Maladies Infectieuses et Tropicales, CHU Souro Sanou, Bobo Dioulasso, Burkina Faso; 13 Department of Medicine, Makerere University College of Health Sciences, Kampala, Uganda; 14 Division of Medical Virology, Department of Pathology, University of Stellenbosch and Tygerberg Academic Hospital, Cape Town, South Africa; 15 Centre de Prise en charge de Recherche et de Formation. CePReF-Aconda-VS, Abidjan, Côte d’Ivoire; 16 Centre Médical de Suivi de Donneurs de Sang/ CNTS/PRIMO-CI, Abidjan, Côte d’Ivoire; 17 Global Hepatitis Programme, HIV Department, World Health Organization, Geneva, Switzerland; 18 Infectious Diseases Institute, Kampala, Uganda; 19 Department of Medicine, Albert Einstein College of Medicine and Montefiore Medical Center, Bronx, New York, USA; 20 ISPED, Université de Bordeaux, Bordeaux, France; 21 INSERM U1219, Bordeaux Population Health, Bordeaux, France; 22 Department of Infectious Diseases, Bern University Hospital, University of Bern, Bern, Switzerland

## Abstract

**Background:**

Approximately 8% of HIV-infected individuals are co-infected with hepatitis B virus (HBV) in sub-Saharan Africa (SSA). Knowledge of HBV status is important to guide optimal selection of antiretroviral therapy (ART) and monitor/prevent liver-related complications. We describe changes in testing practices and management of HBV infection over a 3-year period in HIV clinics across SSA.

**Methods:**

A medical chart review was conducted in large urban HIV treatment centers in Côte d’Ivoire (3 sites), Benin, Burkina Faso, Cameroon, Kenya, Senegal, South Africa, Togo, Uganda and Zambia (1 site each). Of the patients who started ART between 2010 and 2012, 100 per year were randomly selected from each clinic. Demographic, clinical and laboratory information as well as individual treatment histories were collected using a standardized questionnaire. We examined changes over time in the proportion of patients screened for HBV infection (HBV surface antigen [HBsAg]-positivity), identified predictors of HBV testing using logistic regression, and assessed the proportion of patients initiating a tenofovir (TDF)-containing ART regimen.

**Results:**

Overall, 3579 charts of patients initiating ART (64.4% female, median age 37 years) were reviewed in 12 clinics. The proportion of patients screened for HBsAg increased from 17.8% in 2010 to 24.4% in 2012 overall, and ranged from 0.7% in Kenya to 96% in South Africa. In multivariable analyses, age and region were associated with HBsAg screening. Among 759 individuals tested, 88 (11.6%; 95% confidence interval [CI] 9.4–14.1) were HBV-infected, of whom 71 (80.7%) received a TDF-containing ART regimen. HBsAg-positive individuals were twice as likely to receive a TDF-containing first-line ART regimen compared to HBsAg-negative patients (80.7% vs. 40.3%, *p* < 0.001). The proportion of patients on TDF-containing ART increased from 57.9% in 2010 to 90.2% in 2012 in HIV/HBV-co-infected patients (Chi-2 test for trend: *p* = 0.01). Only 114 (5.0%) patients were screened for anti-HCV antibodies and one of them (0.9%, 95% CI 0.02–4.79) had a confirmed HCV infection.

**Conclusions:**

The systematic screening for HBV infection in HIV-positive patients before ART initiation was limited in most African countries and its uptake varied widely across clinics. Overall, the prescription of TDF increased over time, with 90% of HIV/HBV-coinfected patients receiving this drug in 2012.

## Background

In sub-Saharan Africa (SSA), approximately 8% of HIV-infected individuals are hepatitis B surface antigen (HBsAg)-positive [1]. In SSA, most hepatitis B virus (HBV) infections occur before the age of 5 years through household contacts [2] and HBV is the single most important cause of liver disease with attributable fractions of cirrhosis and hepatocellular carcinoma reaching 34% and 47%, respectively [3]. However, in the absence of routine screening in the general population, the majority of infected people do not know their HBV status.

HIV/HBV-coinfected patients are at higher risk of impaired immunological recovery and hepatotoxicity during antiretroviral therapy (ART) compared to HBV-uninfected ones, and overall mortality tends to be higher in the presence of HBV infection [4–6]. Knowledge of HBV status at ART start is important for clinical monitoring and to guide the selection of the initial ART regimen, as tenofovir disoproxil fumarate (TDF) should be combined with lamivudine (3TC) or emtricitabine (FTC) and a non-nucleoside reverse transcriptase inhibitor (NNRTI) in the presence of HBV infection [7, 8]. Knowledge of HBV status has other benefits: it helps to guide the choice of second-line treatment in case of failure of initial ART or drug toxicity; enables HBV vaccination in those non-immune; and in pregnant women allows optimization of prevention of mother-to-child transmission through use of immunoglobulins and antivirals in addition to birth dose of hepatitis B vaccine and infant vaccination.

Although most international and national ART guidelines recommend screening for HBV infection prior to ART initiation, HBV testing uptake has been poor in most resource-limited settings (RLS). In a recent analysis of over 60,000 patients initiating ART in Lusaka, Zambia, the proportion of patients tested for HBV co-infection increased steadily between 2010 and 2012 but remained below 50% overall and screening uptake varied widely across clinics [9]. Taking advantage of a large, international HIV cohort collaboration in SSA, we aimed to describe changes in testing practices related to viral hepatitis over a 3-year period in HIV clinics from ten countries across SSA. Our main objective was to measure HBV and hepatitis C virus (HCV) testing uptake in routine clinical settings. In addition, through standardized chart extractions we investigated the impact of HBV infection on the choice of initial ART regimen in these settings. A further objective was to provide insight into current viral hepatitis testing practices and feasibility challenges in HIV clinics across SSA to inform international guidelines [10].

## Methods

### Study settings

**Fig. 1:**
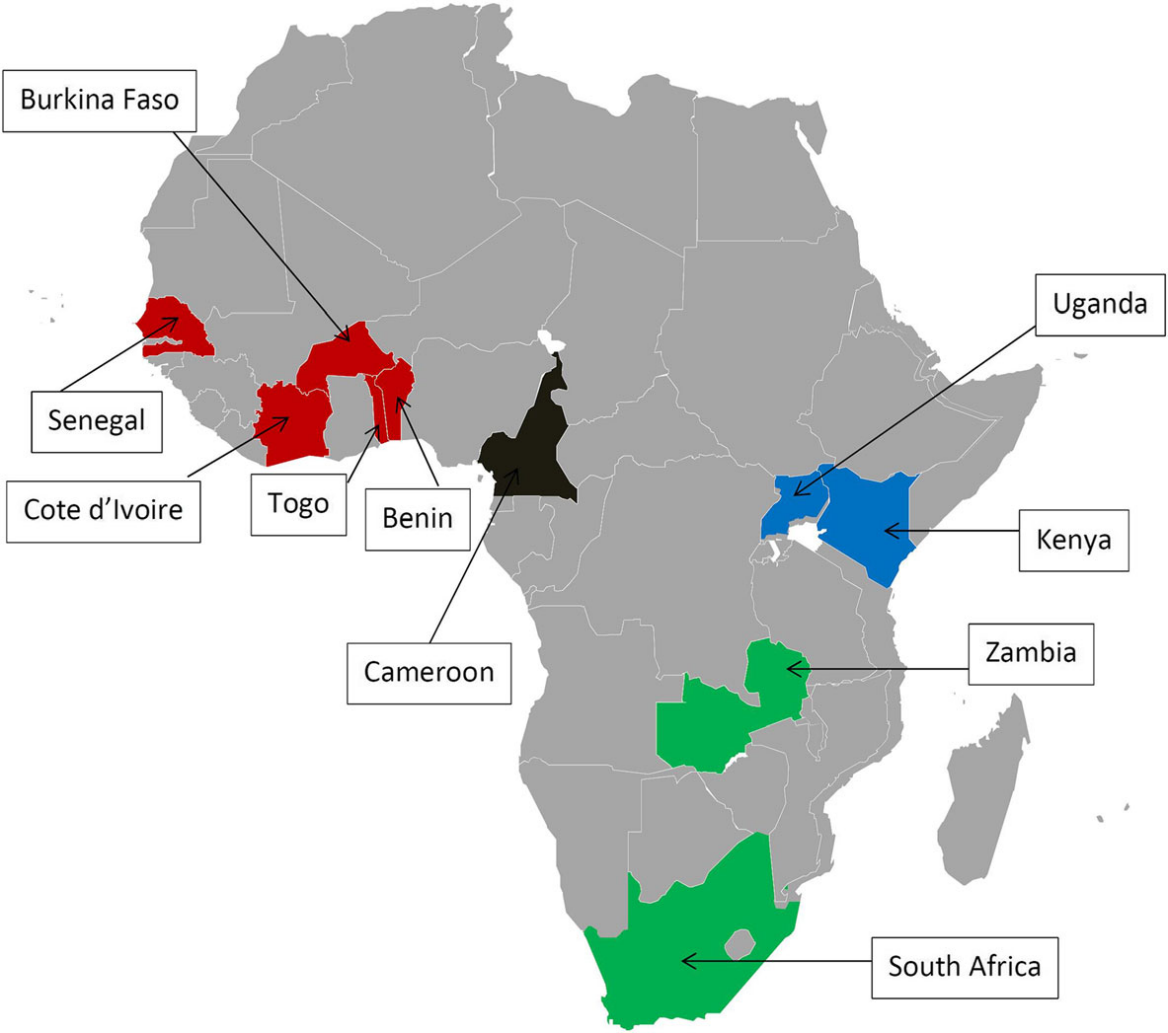
Geographical distribution of participating sites (Red: West Africa; blue: East Africa; black: Central Africa; green: Southern Africa)

We performed a retrospective survey in 12 mostly urban tertiary hospitals from the following countries in SSA: Côte d’Ivoire (3 sites), Benin, Burkina Faso, Cameroon, Kenya, Senegal, South Africa, Togo, Uganda and Zambia (1 site each), as shown in Fig. 1. All clinics were part of the International epidemiologic Databases to Evaluate AIDS (IeDEA) collaboration, a large international research network of HIV cohorts [11]. The study received approval from local ethics committees of each of the ten participating countries:

### Study population and data collection

Among the total number of patients who started ART between 2010 and 2012, we randomly selected a sample of 100 adults (>18 years) initiating ART per year at each clinic, i.e. up to 300 patients per clinic in total. If the number of patients starting ART was smaller than 100 for a given year, all patients were selected for that year. All demographic and HIV-related clinical data were entered into a local database for clinical care, evaluation and reporting purposes, within the IeDEA framework. As information on viral hepatitis screening, monitoring and treatment were not routinely available in the data collected by IeDEA, we performed a chart review using a standardized data collection protocol in all study sites. In addition to data on screening for HBsAg, anti-HCV antibody and any other available HBV and HCV serological and virological tests, we collected information related to ART history, routinely performed laboratory tests and HCV treatment.

### Statistical analyses

Patient characteristics were presented as medians with interquartile range (IQR) for continuous variables and absolute numbers and percentages for categorical ones. The proportion of people tested and the prevalence of HBV infection were reported with 95% confidence intervals (CI) overall and by year of ART initiation and country. The proportion of individuals receiving TDF as part of their initial ART regimen was evaluated by HBsAg status. Differences in proportions of HBsAg-positivity between countries were assessed using Chi-square and Fisher’s exact tests. Changes in screening uptake over time were evaluated with a Chi-square test for trend. We investigated associations between HBsAg testing and sex, age and region (Central Africa, East Africa, West Africa and Southern Africa) using logistic regression. Variables associated with HBsAg screening in univariable regression (*p* < 0.20) were included in multivariable models. All statistical analyses were performed using Stata 9.0 (StataCorp, College Station, TX, USA).

## Results

### Characteristics of clinical sites and patients

A total of 3579 HIV-infected patients were included: 300 per clinic, except in the following sites: Cameroon (*n* = 298), Kenya (*n* = 299), Senegal (*n* = 297) and Uganda (*n* = 285). All clinics were located in urban settings and the large majority of them were tertiary health care facilities. Table 1 summarizes patient characteristics at ART initiation, by country. The proportion of patients from each site included in this study ranged from 1% in Kenya to 91% in Senegal. The median age was 37 years (IQR: 31–44) and about two-thirds of patients (64.4%) were women. For 93.9% of patients, the first-line ART regimen contained two nucleoside reverse transcriptase inhibitors (NRTI) and one NNRTI. Overall, 1477 (41.3%) patients started a TDF-containing ART regimen with the lowest proportion in Benin (13.0%) and the highest in Zambia (88.3%).

**Table 1:** Characteristics of HIV-infected patients at initiation of antiretroviral therapy in ten African countries, 2010–2012 (*N* = 3579)

	Benin	Burkina Faso	Cameroon	Cote d’Ivoire	Kenya	Senegal	South Africa	Togo	Uganda	Zambia
Number of sites	1	1	1	3	1	1	1	1	1	1
Cities	Cotonou	Bobo Dioulasso	Limbé	Abidjan	Eldoret	Dakar	Stellenbosch	Lomé	Kampala	Lusaka
Type of care	Tertiary	Tertiary	Tertiary	Primary / Tertiary	Tertiary	Tertiary	Tertiary	Tertiary	Tertiary	Primary
Number of patients included (proportion of total population followed)	300 (30%)	300 (21%)	298 (~30%)	900 (20%)	299 (1%)	297 (91%)	300 (16%)	300 (13%)	285 (7%)	300 (~10%)
Median age in years (IQR)	35 (30–42)	35 (30–42)	38 (32–44)	38 (33–45)	37 (32–44)	41 (35–49)	38 (33–46)	35 (30–43)	35 (28–42)	37 (31–42)
Female sex (%)	198 (66.0)	216 (72.0)	196 (65.8)	581 (64.6)	190 (63.5)	179 (60.3)	193 (64.3)	197 (65.7)	191 67.0)	162 (54.0)
Type of ART (%)										
2 NRTI +1 NNRTI	298 (99.3)	269 (89.7)	298 (100)	786 (87.3)	291 (97.3)	286 (96.3)	298 (99.3)	287 (95.7)	253 (88.8)	295 (98.3)
3 NRTI	0 (0.0)	0 (0.0)	0 (0.0)	22 (2.5)	0 (0.0)	2 (0.7)	0 (0.0)	0 (0.0)	0 (0.0)	0 (0.0)
2 NRTI +1 PI	2 (0.7)	31 (10.3)	0 (0.0)	92 (10.2)	8 (2.7)	9 (3.0)	2 (0.7)	13 (4.3)	32 (11.2)	5 (1.7)
TDF (%)	39 (13.0)	87 (29.0)	191 (64.1)	303 (33.7)	122 (40.8)	122 (41.1)	243 (81.0)	44 (14.7)	61 (21.4)	265 (88.3)
HBsAg-positivity (%)	5/35 (14.3)	16/76 (21.1)	2/12 (16.7)	10/37 (27.0)	0/2 (0.0)	25/135 (18.5)	13/279 (4.7)	6/30 (20.0)	4/94 (4.3)	7/59 (11.9)

IQR: interquartile range, ART: antiretroviral therapy, NRTI: nucleoside reverse-transcriptase inhibitors, NNRTI: non-nucleoside reverse-transcriptase

Inhibitors, PI: protease inhibitors, TDF: tenofovir; HBsAg: Hepatitis B surface antigen

### Viral hepatitis screening practices

**Fig. 2:**
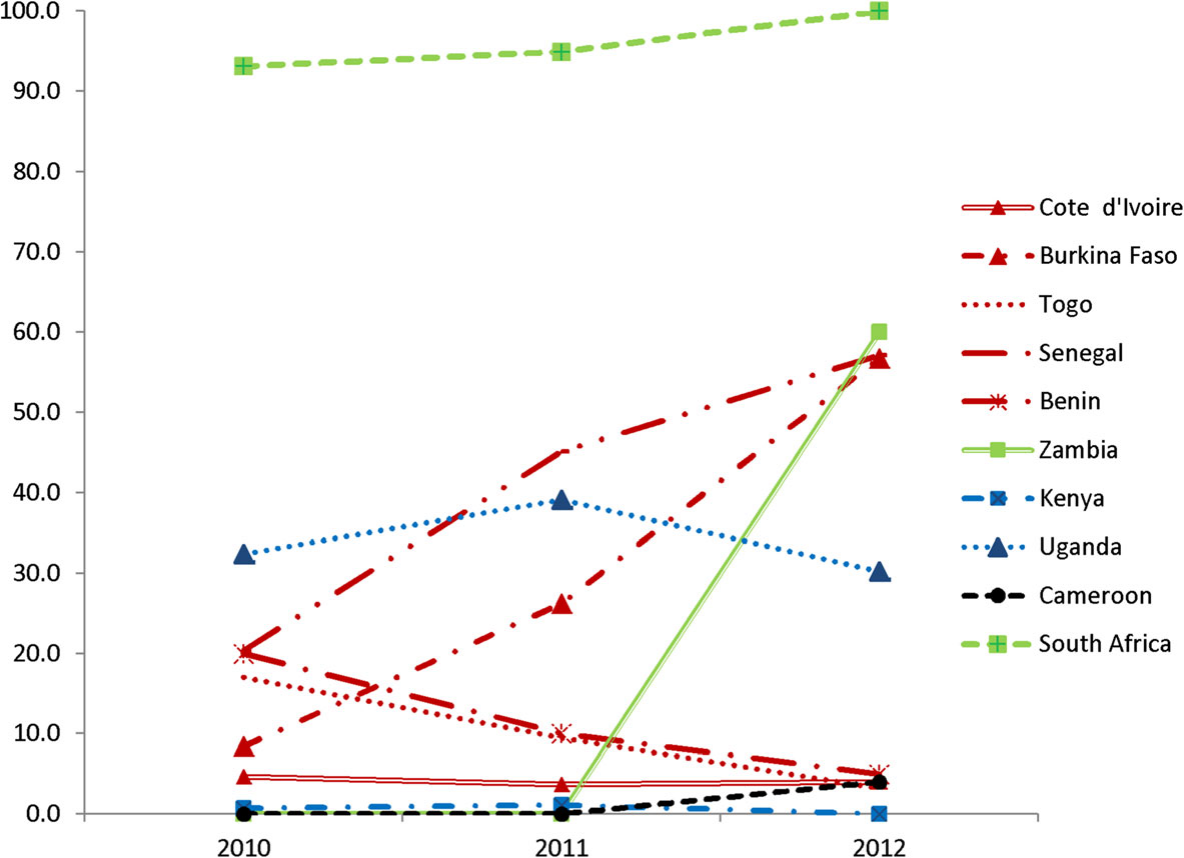
Changes in HBsAg screening over time, by country (2010–2012) (Red: West Africa; blue: East Africa; black: Central Africa; green: Southern Africa)

Overall, 771 patients were tested for HBsAg (21.5%, 95% CI: 20.2–22.9), with a slight increase of this proportion over time: 17.8% of individuals started ART in 2010, 21.9% in 2011 and 24.4% in 2012 (*p* < 0.001; Chi-square test for trend). HBsAg testing practice varied widely across countries, from 0.7% tested 2010–2012 in Kenya to 96.0% tested in South Africa (p < 0.001) (Fig. 2). The proportion tested remained below 50% in most countries during the study period with the exception of South Africa where levels of testing were high throughout, and Zambia, Senegal and Burkina Faso, where the proportion of patients tested increased considerably over time (Fig. 2). Most HBV tests (79.5%) were performed before ART initiation. Fewer than 15 patients were tested in Cameroon, Burkina Faso and Kenya and only the clinic in South Africa had a policy of routine HBsAg testing at HIV diagnosis. No HBsAg-positive patient had a HBV DNA measurement performed to confirm active HBV infection.

In multivariable analyses, patients over 40 years were more likely to be tested for HBsAg than younger patients (adjusted odds ratio [aOR]: 1.20, 95% CI 1.01–1.44), but sex was not associated with HBV screening (Table 2). HBsAg screening was more common in Southern Africa (aOR 33.22, 95% CI 18.23–60.54), East Africa (aOR 4.87, 95% CI 2.62–9.02) and West Africa (aOR 4.20, 95% CI 2.33–7.57) as compared to Central Africa.

**Table 2:** Factors associated with hepatitis B screening among HIV-infected patients in ten African countries, 2010–2012 (N = 3579)

		Univariable analysis			Multivariable analysis		
	n / N (%)	OR	95% CI	*P*	aOR	95% CI	*P*
Age in years (%)				0.09			0.04
≤ 40	469 / 2270 (20.7)	Ref.	–		Ref.	–	
> 40	302 / 1309 (23.1)	1.15	0.98–1.36		1.20	1.01–1.44	
Sex (%)				0.55			
Female	489 / 2303 (21.2)	Ref.	–				
Male	282 / 1276 (22.1)	1.05	0.89–1.24				
African region (%)				<0.001			<0.001
Central	12 / 298 (4.0)	Ref.			Ref.		
West	313 / 2097 (14.9)	4.18	2.32–7.54		4.20	2.33–7.57	
East	98 / 584 (16.8)	4.81	2.59–8.91		4.87	2.62–9.02	
South	348 / 600 (58.0)	32.90	18.07–59.96		33.22	18.23–60.54	

n: number of patients screened for HBV; N: total number of patients; OR: odds ratio; aOR: adjusted odds ratio;

CI: confidence interval; P: *p*-value

Information on HCV testing was available in the files for 2271 individuals (63.4% of the total study sample). Overall, only 114 (5.0%) of these patients were tested for the presence of anti-HCV antibodies. The sites in Kenya, Zambia and Cameroon did not perform any HCV testing and only sites in Côte d’Ivoire (11 patients, 8.9%), Burkina Faso (21 patients, 7.0%), Senegal (19 patients, 6.4%) and South Africa (45 patients, 15.0%) tested more than 10 patients. Only two patients had a positive anti-HCV result, and one of them was a confirmed HCV infection by nucleic acid amplification. Among 114 patients tested for anti-HCV, 110 were also screened for HBsAg. No patients tested positive for both HBsAg and anti-HCV.

### Prevalence of HBsAg and initial treatment

Among 771 patients screened for HBsAg, 12 results were not available in the patient charts (2 in Uganda, 1 in Zambia and 9 in South Africa). Of 759 individuals with HBsAg results, 88 (11.6%; 95% CI 9.4–14.1) had a positive test. The prevalence of HIV/HBV-co-infection was 0% in Kenya, 4.3% in Uganda, 4.7% in South Africa, 11.9% in Zambia, 14.3% in Benin, 16.7% in Cameroon, 18.5% in Senegal, 20.0% in Togo, 21.1% in Burkina Faso and 27.0% in Côte d’Ivoire (*p* < 0.001).

**Fig. 3:**
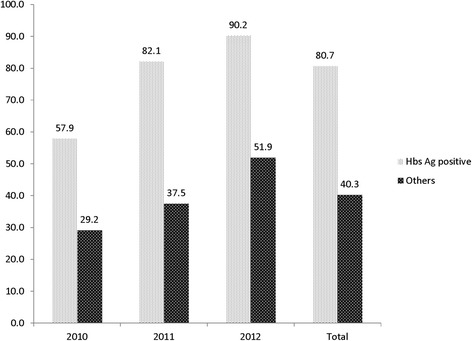
Proportion of patients on tenofovir-based regimens, by year and HBsAg status in ten sub-Saharan African countries, 2010–2012. Others: HbsAg-negative or not screened

HBsAg-positive individuals were twice as likely to receive a TDF-containing first-line ART regimen compared to HBsAg-negative patients (80.7% vs. 40.3%, p < 0.001 Fig. 3). The proportion of patients on TDF-containing ART increased from 57.9% in 2010 to 90.2% in 2012 in HIV/HBV-co-infected patients (Chi-2 test for trend: *p* = 0.01) and from 29.2% in 2010 to 51.9% in 2012 in HBV-uninfected individuals (Chi-2 test for trend: p < 0.001). In West Africa, where TDF was not part of first-line ART options during the study period, 77.4% of HIV/HBV-co-infected individuals received a TDF-containing regimen, whereas this was only the case for 26.9% of HBV-uninfected ones.

## Discussion

Despite the consistent recommendation in national and international HIV guidelines to test all HIV-infected patients for HBV-coinfection before ART initiation, screening uptake was generally poor in this study of 12 large urban tertiary hospitals in SSA. Of over 3500 HIV-infected individuals from ten African countries, less than one-quarter was tested for the presence of HBV infection. In most sites, HBsAg testing uptake increased only minimally between 2010 and 2012, with large differences in the proportion of patients tested across clinics and countries. None of the 88 HIV/HBV-co-infected patients in this sample had an HBV viral load test performed after a positive HBsAg test. However, the majority of HIV/HBV-co-infected patients initiated a standard-of-care TDF-containing ART regimen.

The implementation of HBV testing practices for HIV-infected patients was sub-optimal in the majority of the clinical sites assessed. The South African clinic was the only one to achieve near-complete HBV testing rates throughout the study period, reflecting a local policy of routine HBsAg testing at diagnosis, and a clear improvement in testing uptake over time was only seen in the sites in Burkina Faso, Senegal and Zambia. The limited availability of HBsAg and anti-HCV screening shown in our study reflects a missed opportunity as the population studied is already engaged in HIV care and testing would likely be acceptable to most patients. There are several potential barriers to more widespread adoption of testing for viral hepatitis in SSA, including financial reasons (the costs of rapid tests and nucleic acid testing are rarely covered by national HIV programs or donor agencies); logistical reasons (poor availability of quality-assured rapid tests in most clinics), and lack of clear national guidance on testing strategies [12]. There is also an emerging view that with the increasing use of TDF-based ART as the preferred first-line regimen, the majority of HBV-coinfected patients will receive effective suppressive treatment and HBsAg testing is no longer required. However, knowledge of HBsAg status is particularly important when switching to second-line ART for treatment failure or drug toxicity to ensure that the new regimen continues to be effective against HBV and avoid the risk of flare [13]. Whereas health disparities in access to care for liver diseases in SSA are well documented [14], similar system-related barriers to viral hepatitis testing have been underlined in a recent report for Europe [15]. Thus, there is an urgent need to improve access to simple and cheap diagnostic tests for HBV and HCV infection globally, a goal that can only be achieved by translating international guidelines into national strategies and increased political will and resources [16]. Improvements in blood safety in SSA illustrate that with adequate funding mechanisms and a well-defined global policy high success rates are achievable: the proportion of countries testing at least 95% of blood donations for HBsAg increased from 76% to 94% in the last decade [17].

As expected in settings where HBsAg testing was limited, additional hepatitis diagnostic testing was uncommon. Indeed, due to high cost and limited laboratory capacities in most sites, HBV DNA measurement was unavailable. The use of dried blood spots for HBV viral load measurements [18], and less expensive nucleic acid amplification techniques should improve uptake of confirmatory HBV viral load testing in the future. HCV screening was unavailable in most settings and only performed in highly selected groups of patients. More than 75% of HCV test results came from the cohorts in South Africa, Senegal and Burkina Faso, where access to HCV testing was improved by the presence of specifically trained laboratory personnel. Overall only two patients had a positive anti-HCV test, in line with recent data from Zambia and Mozambique which highlighted the low prevalence of HCV infection in southern Africa [19]. However, as the burden of HCV infection in HIV-positive people varies widely across African sub-regions [20], HCV testing strategies should be tailored to the local epidemiological context, including routine testing in high prevalence settings and focused testing in HIV-infected high-risk groups such as persons who inject drugs, men who have sex with men, sex workers and prisoners, as well as children of HIV/HCV-co-infected women.

The prevalence of HBV-infection (11.6%) in our study probably overestimated the true population prevalence among people living with HIV: several clinics had more than 20% of patients with a positive HBsAg test, most likely because patients with clinical features of liver disease were more likely to be tested than asymptomatic patients. This is also reflected by the lower HBV-prevalence found in South Africa, where systematic screening was performed. Importantly, the proportion of HIV/HBV-co-infected individuals who received a standard-of-care TDF-containing regimen increased over time. Considering that over two-thirds of HBV infections were diagnosed in West African sites where TDF was not part of the preferred first-line ART regimens during the study period, it is encouraging to note that by 2012, 90% of HBV-infected patients had TDF included in their initial ART regimen, irrespective of clinic or country. Conversely, HBsAg-negative patients were less likely to receive TDF, reflecting the national treatment guidelines during the study period. By 2012, 80% of low-and middle-income countries globally had adopted the 2010 World Health Organization (WHO) guidelines and began the transition away from stavudine to TDF or zidovudine (AZT) for all new ART initiations [21]. However, progress in the uptake of TDF as part of the preferred first-line ART has been variable across countries: whereas Zambia started to include TDF in first-line ART in 2007, many West African countries were still using AZT in first-line ART at the end of 2012. In fact, most countries in West Africa only started the implementation of TDF in first-line ART in 2015.

This is the largest study to date assessing HBV and HCV screening practices in routine clinical HIV care settings in SSA. It was nested within an established observational data collection platform (the IeDEA network) and utilized standardized data collection instruments at all sites for chart review. Unfortunately, we could not obtain more detailed information on demographic factors such as education level or employment, as these data were not reported identically in the charts of most clinics. Socioeconomic factors could well have played a role in access to HBV and HCV screening in our study, especially in settings where no systematic testing was performed. A further limitation of our study was the absence of data on the reasons for HBV testing for the majority of patients, except for the South African clinic which had a documented policy of screening all patients during the study period. As a consequence, we could not determine the proportion of patients who were tested due to clinical signs of liver disease, which potentially led to an over-estimation of HBsAg prevalence. In addition, we may have under-estimated the proportion of HBV-coinfected patients in our study as we would not have diagnosed patients with occult HBV infection, which is expected to be present in 10%–15% of HIV-infected individuals in SSA [22, 23]. Finally, as only large urban clinics were selected for this study, our results may not be generalizable to rural areas. Indeed, HBV and HCV testing is likely to be even more limited in rural settings.

In summary, our results highlight the heterogeneity in HBsAg testing and the near-absence of HCV testing for HIV-infected individuals in SSA. As the availability of TDF for long-term treatment of HBV infection and direct-acting antivirals for HCV infection is increasing globally, early diagnosis of HBV and, in specific risk groups, of HCV infections is of utmost importance. The WHO hepatitis testing guidelines recommend focused testing for HBV and HCV in most affected populations that include HIV-infected persons [10]. They also recommend that hepatitis testing should make use of existing opportunities and infrastructure for testing that include health facility and community-based testing for HIV. The availability of polyvalent platforms will allow increasingly for multiplex testing for HBV, HCV, HIV and syphilis. HIV programs should consider adopting HBsAg testing uptake as a metric for program evaluation and surveys similar to this one should be repeated regularly to monitor progress, including in rural areas. Finally, in order to improve current testing practices, more efforts are needed to understand the main reasons for the limited uptake of HBV and HCV diagnostic practices in many low-income settings.
